# Socioeconomic inequalities in the prevalence of biomarkers of cardio-metabolic disease in South Korea: Comparison of the Health Examinees Study to a nationally representative survey

**DOI:** 10.1371/journal.pone.0195091

**Published:** 2018-04-18

**Authors:** Sujin Kim, Juhwan Oh, Jongho Heo, Hwa-Young Lee, Jong-Koo Lee, S. V. Subramanian, Daehee Kang

**Affiliations:** 1 Korea Institute for Health and Social Affairs, Sejong city, South Korea; 2 Institute for Health and Environment, Seoul National University, Seoul, South Korea; 3 JW Lee Center for Global Medicine, Seoul National University College of Medicine, Seoul, South Korea; 4 Department of Global Health and Population, Harvard T.H. Chan School of Public Health, Boston, Massachusetts, United States of America; 5 Department of Family Medicine, Seoul National University College of Medicine, Seoul, South Korea; 6 Department of Social and Behavioral Sciences, Harvard T.H. Chan School of Public Health, Boston, Massachusetts, United States of America; 7 Department of Preventive Medicine, Seoul National University College of Medicine, Seoul, South Korea; 8 Department of Biomedical Sciences, Seoul National University Graduate School, Seoul, South Korea; 9 Cancer Research Institute, Seoul National University, Seoul, South Korea; 10 Institute of Environmental Medicine, Seoul National University Medical Research Center, Seoul, South Korea; Boston University, UNITED STATES

## Abstract

**Background/Objectives:**

This study aimed to examine socioeconomic inequalities in the prevalence of biomarkers of cardiovascular disease and diabetes in the newly developed large-scale genomic cohort study of Korean adults, the Health Examinees-Gem (HEXA-G), with a comparison of the nationally representative cross-sectional study, the Korea National Health and Nutrition Examination Survey (K-NHANES).

**Subjects/Methods:**

Using the HEXA-G and the K-NHANES from 2007–2012, we analyzed the age-adjusted relative risk (RR) and prevalence of enlarged waist circumference (EWC), elevated triglycerides (ET), low HDL cholesterol (LHC), elevated blood pressure (EBP) and elevated blood glucose (EBG) by income and educational groups for adults at age 40–69.

**Results:**

For men, the prevalence of risk factors was similar across different income and educational groups (p>0.1), and between the K-NHANES and the HEXA-G. Among five risk factors, EBG showed the greatest discrepancy by 7 to 11 percentage points (i.e., the prevalence of 0.43 and 0.36 for college graduates, respectively, in K-NHANES and HEXA-G). For women, socioeconomic inequalities appeared for the five risk factors. Prevalence of risk factors was mostly lower in the HEXA-G than the K-NHANES, by approximately 11.0 percentage points. Especially, the discrepancy between K-NHANES and HEXA-G was largest in EBG (i.e., the prevalence of 0.31 and 0.20 for the lowest income groups, respectively).

**Conclusion:**

The HEXA-G shows broadly similar socioeconomic inequality in prevalence of cardio-metabolic risk factors to the nationally representative sample with more modest socioeconomic inequality among women in the HEXA-G than the K-NHANES.

## Introduction

South Korea has experienced a rapid economic development and westernization. It accompanies environmental changes such as an abundance of high-calorie foods and a decrease in physically demanded work. This has led a sharp increase in chronic diseases such as cardiovascular disease and diabetes.[1–3] In particular, it is a concern that those environmental changes are more likely to affect individuals in socioeconomically disadvantaged background than those advantaged socioeconomically.[4–8] Thus, there is a growing interest in understanding the roles of environmental changes, social factors and genes, and interaction effects among them.[9, 10]

In Korea, the Korea National Health and Nutrition Examination Survey (K-NHANES) is a nationally representative repeated cross-sectional survey, designed to assess the health and nutritional status of Koreans and to monitor trends in health risk factors and the prevalence of major chronic diseases.[11] Although the cross-sectional survey plays a significant role as an ongoing surveillance system to provide timely health statistics based on annual survey, it has a limitation in assessing causal effects of risk factors. In this context, a large-scale genomic cohort, the Health Examinees-Gem (HEXA-G) Study, was established based on the existing health examination system of the Korea National Health Insurance Service (NHIS), which provides biannual health examinations to all Korean adults over the age of 40.[12, 13]

The HEXA-G is expected to facilitate close examination of environmental change, socioeconomic factors and genomic risk factors, and development of more comprehensive preventive strategies for chronic diseases.[12, 13] Nevertheless, since the HEXA-G collects information from individuals who voluntarily participate in health examinations, it is important to ascertain how well the data represent the general population of South Korea. To better understand how the findings of the HEXA-G can be applied to national populations from which they were derived, it is essential to understand how similar the HEXA-G sample is to the population. One way of validation is to compare the prevalence obtained from the HEXA-G to the corresponding rate from the K-NHANES, a nationally representative survey. In this line, the purposes of this study were to: (1) determine associations of individual-level socioeconomic status with prevalence of risk factors related to cardiovascular disease and diabetes in the newly developed large-scale genomic cohort study; and (2) for comparison purposes, estimate the national prevalence of biomarkers of cardiovascular disease and diabetes using a nationally representative cross-sectional survey.

## Materials and methods

### Ethics statement

A consent form was filled by all of the participants before participation of the survey. The study was approved by the Institutional Review Board (IRB) of Seoul National University Hospital, Seoul, Korea (IRB NO. 0608-018-179).

### The HEXA-G

This study used baseline data of HEXA-G conducted in South Korea from 2004–2013. The HEXA-G was updated from the previously published HEXA studies, a large-scale community-based prospective cohort for people aged 40–69 years old that were recruited in 38 health examination centers and training hospitals in 8 of 16 regions of South Korea.[13] Of the original 38 sites, HEXA-G excluded (1) 8 sites that only participated in the pilot study from 2004 to 2006 (n = 9370), (2) 8 sites that did not meet the HEXA biospecimen quality control criteria (i.e., different testing protocols) (n = 12,205), and (3) 5 sites that have participated in the study for less than 2 years (n = 8799). In the new HEXA-G data, a total of 139,348 participants remained. Information on socio-demographic characteristics, medical history and medication usage, and health behavior was collected with a structured questionnaire. Skillful medical staff conducted physical examinations and collection and analysis of biological specimens. Laboratory tests for blood were conducted by central laboratory. Further information on the HEXA-G can be found elsewhere.[12] The current study used 113,605 adults the HEXA-G from 2007 to 2012.

Data were collected following a standardized study protocol that was approved by the Ethics Committee of the Korean Health and Genomic Study of the Korean National Institute of Health and institutional review boards from all participating hospitals. All study participants voluntarily signed a consent form before entering the study.

### K-NHANES

K-NHANES from 2007 to 2012 were used for the present study. The K-NHANES is a nationally representative cross-sectional survey administered by the Korea Centers for Disease Control and Prevention. For examining health and nutritional status of Koreans, the survey collects detailed information on socio-demographic characteristics, health behaviors, chronic diseases, healthcare utilization, and indicators of some biological state or condition. The survey was first implemented in 1998 and has conducted annually since 2007. It composes of non-institutionalized Korean citizens residing in Korea. Participants are selected based on a multi-stage clustered probability sampling design. The K-NHANES provides information on sampling design, so statistics representing the entire Korean population can be estimated by adjusting for complex survey designs, survey non-response and post-stratification.[11] More information on the K-NHANES can be found at http://K-NHANES.cdc.go.kr/K-NHANES/eng.

### Outcome measures

The outcome measures included waist circumference, systolic and diastolic blood pressure, fasting plasma glucose, HDL cholesterol and triglycerides. We assessed the presence (or absence) of each risk factor based on the National Cholesterol Education Program Adult Treatment Panel III (NCEP-ATP III) criteria.[2, 14] Enlarged waist circumference (in cm) was defined as waist circumference ≥90 cm for men and ≥85 cm for women. Elevated triglycerides represented fasting triglyceride levels ≥150 mg/dL or specific treatment for this lipid abnormality. Low HDL cholesterol was defined as HDL cholesterol <40mg/dL for men and <50mg/dL for women or specific treatment for this lipid abnormality. Elevated blood pressure represented systolic blood pressure ≥130 mm Hg and/or diastolic blood pressure ≥85 mm Hg or specific treatment for this hypertension. Elevated blood glucose was defined as fasting plasma glucose ≥100 mg/dL or specific treatment for this glucose abnormality.

### Independent variable of interest

Socioeconomic status was assessed with household income and individual education. Since household income was measured in broad categories (eight groups) in the HEXA-G, it was not possible to measure household equivalised income. We categorized household income to four groups ensuring each group to have even number of observations in the HEXA-G: <2, 2–3, 3–4, and ≥ 4 million Korean Won per month. Individual education was categorized into four groups: elementary school, middle school graduates, high school graduates and college graduates. For comparison, we categorized income and education groups in K-NHANES in the same way.

### Statistical analysis

For the K-NHANES and HEXA-G, the relative risk (RR) of risk factors comparing lower to higher socioeconomic groups were calculated by using Poisson regression models adjusting for age (40–49, 50–59, 60–69). Next, the prevalence were age-standardized to the 2010 Korean population. To be representative of the Korean population, we estimated RR and prevalence in accordance with the survey sample design such as sampling weight and cluster for K-NHANES. Since HEXA-G is collected based on hospitals, we estimated cluster standard errors. SAS version 9.3 and Stata version 12.0 were used for all analyses.

## Results

### Enlarged waist circumference (EWC)

#### Men

**Fig 1:**
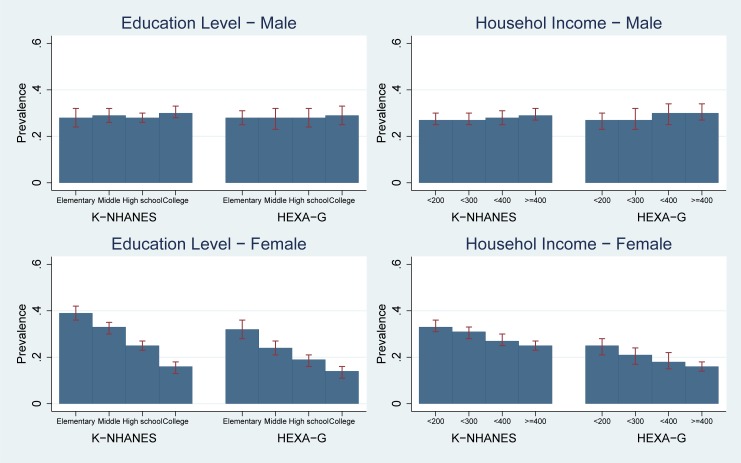
Age-standardized prevalence: Enlarged waist circumference. K-NHANES: Korea National Health and Nutrition Examination Survey; HEXA-G: Health Examinees-Gem. Enlarged waist circumference ≥ 90 in males and ≥ 85 in females.

While the risk of EWC was lower in elementary school graduates than college graduates in K-NHANES (prevalence: 0.28 [95%CI 0.24–0.32] vs. 0.30 [95%CI 0.28–0.33]), HEXA-G showed the similar risk between the two groups (0.28 [95%CI 0.25–0.31] vs. 0.29 [95%CI 0.25–0.33]). With regard to income groups, the prevalence of EWC in K-NHANES were similar between the lowest and highest income groups (0.27 [95%CI 0.25–0.30] vs. 0.29 [95%CI 0.27–0.32]) whereas the rates in HEXA-G showed lower in the lowest than highest income groups (prevalence: 0.27 [95%CI 0.23–0.30] vs. 0.30 [95%CI 0.27–0.34]). The risk of EWC was not concentrated in the low socio-economic groups for both HEXA-G and K-NHANES. The differences in prevalence between HEXA-G and K-NHANES were less than two percentage points (Fig 1, Table A in S1 File).

#### Women

Women with low levels of education were found to have higher likelihood of EWC than college graduates in both K-NHANES and HEXA-G (prevalence in K-NHANES: 0.39 [95%CI 0.36–0.42] vs. 0.16 [95%CI 0.13–0.18] in the lowest and highest education groups; HEXA-G: 0.32 [95%CI 0.28–0.36] vs. 0.14[95%CI 0.11–0.16]). Women in low-income groups had higher risk of EWC than those in high-income group in K-NHANES and HEXA-G (prevalence in K-NHANES: 0.33 [95%CI 0.31–0.36] vs. 0.25 [95%CI 0.23–0.27] in the lowest and highest income groups; HEXA-G: 0.25 [95%CI 0.21–0.28] vs. 0.16 [95%CI 0.14–0.18]). Both HEXA-G and K-NHANES showed higher risk of EWC in low socio-economic groups. The differences in prevalence between HEXA-G and K-NHANES were between two and ten percentage points (Fie 1, Table A in S1 File).

### Elevated triglycerides (ET)

#### Men

**Fig 2:**
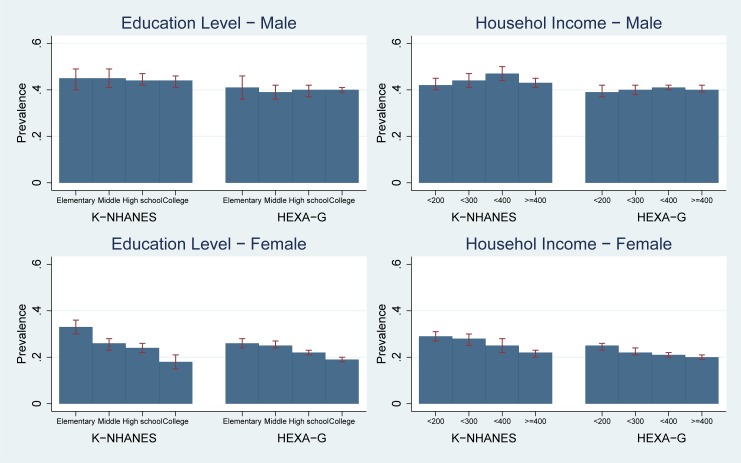
Age-standardized prevalence: Elevated triglycerides. K-NHANES: Korea National Health and Nutrition Examination Survey; HEXA-G: Health Examinees-Gem. Elevated triglycerides ≥ 150 mg/dL (1.7 mmol/L) or specific treatment for this lipid abnormality.

In K-NHANES and HEXA-G, risk of ET was similar across education groups (prevalence in K-NHANES: 0.45 [95%CI 0.40–0.49] for the lowest vs. 0.44 [95%CI 0.41–0.46] for the highest; HEXA-G: 0.41 [95%CI 0.36–0.46] vs. 0.40 [95%CI 0.39–0.41]). The prevalence were similar across different income groups (K-NHANES: 0.42 [95%CI 0.40–0.45] for the lowest vs. 0.43 [95%CI 0.41–0.45] for the highest; HEXA-G: 0.39 [95%CI 0.37–0.42] vs. 0.40 [95%CI 0.39–0.42]). There was no socioeconomic inequality in the risk of ET for both HEXA-G and K-NHANES. The differences in prevalence between HEXA-G and K-NHANES were three to six percentage points (Fig 2, Table B in S1 File).

#### Women

K-NHANES and HEXA-G showed women with the low levels of education had higher likelihood of ET than college graduates (prevalence in K-NHANES: 0.33 [95%CI 0.30–0.36] for the lowest vs. 0.18 [95%CI 0.15–0.21] for the highest; HEXA-G: 0.26 [95%CI 0.24–0.28] vs. 0.19, [95%CI 0.18–0.20]). In addition, income was negatively related to the risk of ET (prevalence in K-NHANES: 0.29 [95%CI 0.27–0.31] for the lowest vs. 0.22 [95%CI 0.20–0.23] for the highest; HEXA-G: 0.25 [95%CI 0.23–0.26] vs. 0.20 [95%CI 0.19–0.21]). Both HEXA-G and K-NHANES showed higher risk of EWC in low socio-economic groups. Differences in prevalence between HEXA-G and K-NHANES were less than seven percentage points (Fig 2, Table B in S1 File).

### Low HDL cholesterol (LHC)

#### Men

**Fig 3:**
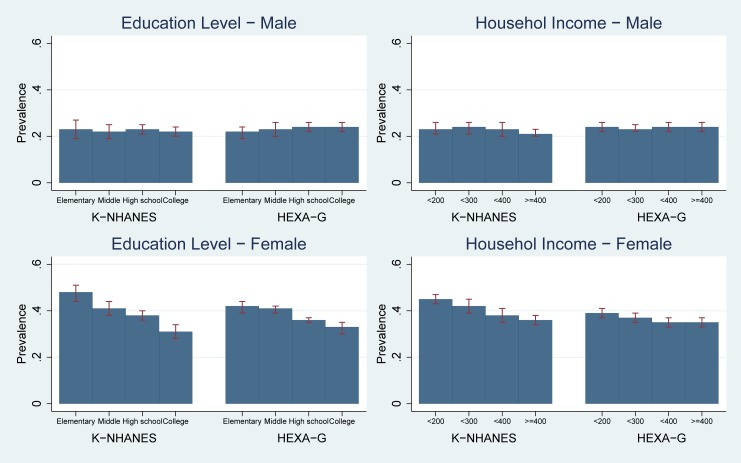
Age-standardized prevalence: Low HDL cholesterol. K-NHANES: Korea National Health and Nutrition Examination Survey; HEXA-G: Health Examinees-Gem. Low HDL cholesterol < 40 mg/dL (1.03 mmol/L) in males, < 50 mg/dL (1.29 mmol/L) in females or specific treatment for this lipid abnormality.

Analyses based on K-NHANES and HEXA-G showed risk of LHC in high-education group did not differ from low-education group (prevalence in K-NHANES: 0.23 [95%CI 0.19–0.27] for the lowest vs. 0.22 [95%CI 0.20–0.24] for the highest; HEXA-G: 0.22 [95%CI 0.19–0.24] vs. 0.24 [95%CI 0.22–0.26]). The likelihood of LHC was not related to income level (prevalence in K-NHANES: 0.23 [95%CI 0.21–0.26] for lowest vs. 0.21 [95%CI 0.20–0.23] for the highest; HEXA-G: 0.24 [95%CI 0.22–0.26]). Both HEXA-G and K-NHANES did not show socioeconomic inequality in the risk of LHC. Differences in prevalence between HEXA-G and K-NHANES were less than three percentage points (Fig 3, Table C in S1 File).

#### Women

K-NHANES and HEXA-G showed education level was negatively related to likelihood of LHC (prevalence in K-NHANES: 0.48 [95%CI 0.44–0.51] for elementary school vs. 0.31 [95%CI 0.28–0.34] for college graduates; HEXA-G: 0.42 [95%CI 0.39–0.44] vs. 0.33 [95%CI 0.30–0.35]). Women in low-income groups had higher risk of LHC, compared to the highest income group (prevalence in K-NHANES: 0.45 [95%CI 0.43–0.47] for the lowest vs. 0.36 [95%CI 0.34–0.38] for the highest; HEXA-G: 0.39 [95%CI 0.37–0.41] vs. 0.35 [95%CI 0.33–0.37]). Low socio-economic groups had higher risk of LHC in both HEXA-G and K-NHANES. The differences in prevalence between HEXA-G and K-NHANES were less than six percentage points (Fig 3, Table C in S1 File).

### Elevated blood pressure (EBP)

#### Men

**Fig 4:**
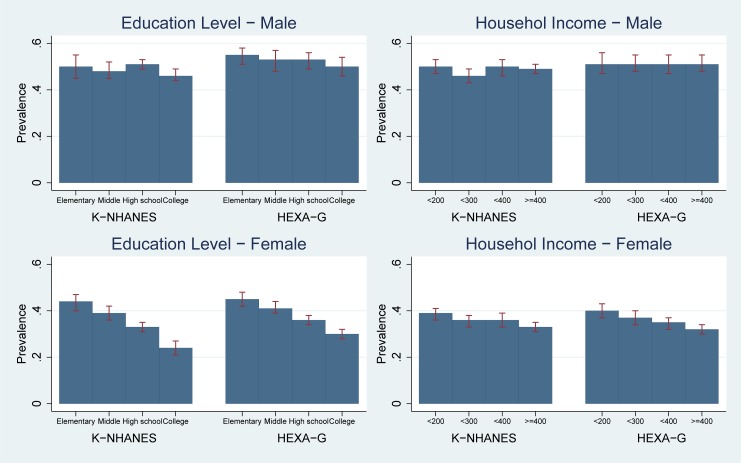
Age-standardized prevalence: Elevated blood pressure. K-NHANES: Korea National Health and Nutrition Examination Survey; HEXA-G: Health Examinees-Gem. Elevated blood pressure (BP) systolic BP ≥ 130 or diastolic BP ≥ 85 mm Hg or specific treatment for this hypertension.

K-NHANES and HEXA-G showed risk of EBP was not different between the lowest and highest levels of education (prevalence in K-NHANES: 0.50 [95%CI 0.45–0.55] for the lowest vs. 0.46 [95%CI 0.44–0.49] for the highest; HEXA-G: 0.55 [95%CI 0.51–0.58] vs. 0.50 [95%CI 0.46–0.54]). Men in the lowest and highest income groups had similar risks of EBP (prevalence in K-NHANES: 0.50 [95%CI 0.47–0.53] for the lowest vs. 0.49 [95%CI 0.47–0.51] for the highest; HEXA-G: 0.51 [95%CI 0.47–0.56] vs. 0.51 [95%CI 0.48–0.55]). There was no socio-economic inequality for both K-NHANES and HEXA-G. The differences in prevalence between HEXA-G and K-NHANES were less than five percentage points (Fig 4, Table D in S1 File).

#### Women

K-NHANES and HEXA-G showed women with the lower levels of education had higher prevalence of EBP than college graduates (prevalence in K-NHANES: 0.44 [95%CI 0.40–0.47] for elementary school vs. 024 [95%CI 0.21–0.27] for college graduates; HEXA-G: 0.45 [95%CI 0.42–0.48] vs. 0.30 [95%CI 0.28–0.32]). Women in low-income groups had higher risk of EBP than those in high-income group (prevalence in K-NHANES 0.39 [95%CI 0.36–0.41] for the lowest vs. 0.33 [95%CI 0.31–0.35] for the highest; HEXA-G: 0.40 [95%CI 0.37–0.43] vs. 0.32 [95%CI 0.30–0.34]). Both HEXA-G and K-NHANES showed higher risk of EBP in low socio-economic groups. The differences in prevalence between HEXA-G and K-NHANES were less than six percentage points (Fig 4, Table D in S1 File).

### Elevated blood glucose (EBG)

#### Men

**Fig 5:**
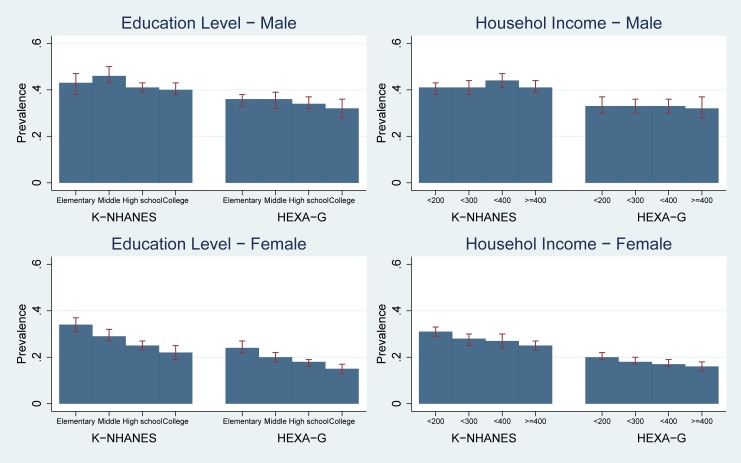
Age-standardized prevalence: Elevated blood glucose. K-NHANES: Korea National Health and Nutrition Examination Survey; HEXA-G: Health Examinees-Gem. Elevated fasting plasma glucose (FPG) ≥ 100 mg/dL (5.6 mmol/L) or specific treatment for this glucose abnormality.

Results from K-NHANES and HEXA-G showed there were no differences in the risk of EBG between the lowest and highest education groups (prevalence in K-NHANES: 0.43 [95%CI 0.38–0.47] for elementary school vs. 0.40 [95%CI 0.38–0.43] for college graduates; HEXA-G: 0.36 [95%CI 0.33–0.38] vs. 0.32 [95%CI 0.28–0.36]). In addition, risk of EBG was not related to income (prevalence in K-NHANES: 0.41 [95%CI 0.38–0.43] for the lowest vs. 0.41 [95%CI 0.39–0.44] for the highest; HEXA-G: 0.33 [95%CI 0.30–0.37] vs. 0.32 [95%CI 0.28–0.37]). Both HEXA-G and K-NHANES did not show socio-economic inequality in risk of EBG. The differences in prevalence between HEXA-G and K-NHANES ranged between seven and 11 percentage points (Fig 5, Table E in S1 File).

#### Women

K-NHANES and HEXA-G showed women with low-education level had higher likelihood of EBG than college graduates (prevalence in K-NHANES: 0.34 [95%CI 0.31–0.37] for elementary school vs. 0.22 [95%CI 0.19–0.25] for college graduates; HEXA-G: 0.24 [95%CI 0.22–0.27] vs. 0.15 [95%CI 0.13–0.17]). Women in low-income groups had higher risk of EBG than those in high-income group (prevalence in K-NHANES: 0.31 [95%CI 0.29–0.33] for the lowest vs. 0.25 [95%CI 0.23–0.27] for the highest; HEXA-G: 0.20 [95%CI 0.19–0.22] vs. 0.16 [95%CI 0.14–0.18]). Both HEXA-G and K-NHANES showed higher risk of EBG in low socio-economic groups. The differences in prevalence between HEXA-G and K-NHANES ranged between seven and 11 percentage points (Fig 5, Table E in S1 File).

## Discussion

This study has two major findings. First, we found relatively good concordance between prevalence of cardio-metabolic risk factors in the HEXA-G and the K-NHANES. Age-adjusted prevalence of each risk factor in the HEXA-G was similar to the K-NHANES, especially for men. For women, prevalence of risk factors was lower in the HEXA-G than the K-NHANES, by approximately 11.0 percentage points at maximum. Second, inequality trend across different socioeconomic status was also consistent between the two studies. Cardio-metabolic risk factors were more concentrated in low socioeconomic status among women, but not men, in both K-NHANES and HEXA-G although the socioeconomic inequalities were greater in the K-NHANES than the HEXA-G.

More concordance between the HEXA-G and the K-NHANES in male than female participants may be related to the fact that HEXA-G is based on health examinees. According to the Occupation Safety and Health Acts in Korea, the penalty has been imposed on both employees and employers since 2003 when the employees do not take regular health check-up. The employment rate is much higher in men than women in Korea (respectively, 81.68 and 41.50 in HEXA; 84.96, 55.45 in K-NHANES), which may lead to lower selection of healthy or health-conscious people in male than female participants, and in turn may reduce discrepancy in prevalence of risk factors between the HEXA-G and the K-NHANES. In contrast, women with greater health consideration and better health behavior are more likely to participate in health examinations and in turn the HEXA-G. This may lead to more modest prevalence of cardio-metabolic risk factors among women.

The inverse association between socioeconomic status and cardio-metabolic risk factors is repeatedly reported in previous work although causal relationship and mechanisms are less known. Explanation for the relationship includes different health behavior across different socioeconomic groups. Low socioeconomic status is associated with smoking,[15] physical inactivity,[16] and unhealthy diet,[17] which are associated with components of cardio-metabolic risk factors.[18] In addition, socioeconomic difference in awareness of health and health-care access may lead to different treatment for hypertension, abnormal blood glucose, and hyperglycemia.[19, 20]

Our findings, more apparent socioeconomic inequality in women than men, are consistent with previous work. Studies that analyzed socioeconomic inequality in cardio-metabolic risk factors in Korea have found similar patterns.[3, 4, 21] The gender heterogeneities in the association of socioeconomic status with cardio-metabolic risk factors were observed in other Asian countries such as Taiwan and China[22, 23] and Western countries such as France and the US.[24, 25] Probably, social norm and culture are related to more apparent socioeconomic inequality in women.[26] For example, obesity is stigmatized more highly in women than men,[27] which is a component of cardio-metabolic risk factors. Women are more likely to have beliefs on the importance of healthy dietary behaviors, but difference in available resources, which are related to socioeconomic status, may lead to great variation in women.[28, 29] In addition, women of higher socioeconomic status may be more knowledgeable about their health and fitness. They therefore may consume healthy food, engage in regular exercise, and check their physical condition periodically. In addition, obesity may limit upward social mobility more so in women than men.[30] In contrast, Korean men of higher socioeconomic status have a more sedentary lifestyle and many opportunities to consume richer foods and alcohol beverages but less opportunity to engage in physical labor.[31] Future research focused on identifying mechanisms responsible for gender differences in the relation between socioeconomic status and cardio-metabolic risk factors will provide better knowledge of the potential pathways.

Representativeness of the population may not be a critical issue in scientific studies.[32] Causal relationship could be established in cohort studies by using sufficient measurements and adjusting for potential confounders, and could be generalized by understanding mechanisms of the relationship.[32, 33] Nevertheless, an examination of the representativeness of a cohort helps understand the prevalence of disease or exposures in the population and evaluate unbiased exposure-outcome relationships.[33]

In conclusion, the HEXA-G shows broadly similar socioeconomic inequality in prevalence of cardio-metabolic risk factors to the K-NHANES, a nationally representative sample although socioeconomic inequality among women appeared more modest in the HEXA-G than the K-NHANES. The HEXA-G is expected to provide empirical evidence on causal relationship between cardio-metabolic risk factors and socioeconomic factors if the data are continuously accumulated. In the future, when socioeconomic inequality in cardio-metabolic risk factors is investigated using the HEXA-G, the findings should be interpreted considering the discrepancy observed in the current study.

## Supporting information

S1 File Age-adjusted relative risk (RR) and age-standardized prevalence. (DOCX)
