# Supplementary material for: Socioeconomic inequalities in the prevalence of biomarkers of cardio-metabolic disease in South Korea: Comparison of the Health Examinees Study to a nationally representative survey
Source: PLoS One. 2018 Apr 18;13(4):e0195091. doi: 10.1371/journal.pone.0195091 (PMC5906014; doi:10.1371/journal.pone.0195091)
Supplement: S1 File — (DOCX) [file pone.0195091.s001.docx]

**Supplementary Information. Age-adjusted relative risk (RR) and age-standardized prevalence**

Table A. Age-adjusted relative risk (RR) and age-standardized prevalence: Enlarged waist circumference ^1)^

|  | K-NHANES^2)^ | | | | | HEXA-G | | | | |
| --- | --- | --- | --- | --- | --- | --- | --- | --- | --- | --- |
|  | N | RR | 95% CI  (lower, upper) | Prevalence | 95% CI  (lower, upper) | N | RR | 95% CI  (lower, upper) | Prevalence | 95% CI  (lower, upper) |
| *Male* |  |  |  |  |  |  |  |  |  |  |
| Elementary school | 1,632 | 0.85 | (0.75 - 0.97) | 0.28 | (0.24 - 0.32) | 3,413 | 1.00 | (0.89 - 1.12) | 0.28 | (0.25 - 0.31) |
| Middle school graduates | 1,378 | 0.95 | (0.84 - 1.09) | 0.29 | (0.26 - 0.32) | 4,887 | 0.99 | (0.89 - 1.11) | 0.28 | (0.23 - 0.32) |
| High school graduates | 2,710 | 0.91 | (0.82 - 1.01) | 0.28 | (0.26 - 0.30) | 13,432 | 0.99 | (0.90 - 1.09) | 0.28 | (0.24 - 0.32) |
| College graduates | 2,315 | 1.00 |  | 0.30 | (0.28 - 0.33) | 16,941 | 1.00 |  | 0.29 | (0.25 - 0.33) |
|  | 8,035 |  |  |  |  | 38,673 |  |  |  |  |
| Income<200 | 2,586 | 0.92 | (0.83 - 1.03) | 0.27 | (0.25 - 0.30) | 9,962 | 0.89 | (0.80 - 0.98) | 0.27 | (0.23 - 0.30) |
| Income<300 | 1,479 | 0.91 | (0.80 - 1.03) | 0.27 | (0.25 - 0.30) | 8,823 | 0.90 | (0.79 - 1.02) | 0.27 | (0.23 - 0.32) |
| Income <400 | 1,238 | 0.98 | (0.86 - 1.11) | 0.28 | (0.25 - 0.31) | 7,989 | 0.97 | (0.88 - 1.07) | 0.30 | (0.25 - 0.34) |
| Income ≥400 | 2,769 | 1.00 |  | 0.29 | (0.27 - 0.32) | 10,110 | 1.00 |  | 0.30 | (0.27 - 0.34) |
|  | 8,072 |  |  |  |  | 36,884 |  |  |  |  |
| *Female* |  |  |  |  |  |  |  |  |  |  |
| Elementary school | 3,963 | 2.54 | (2.15 - 3.01) | 0.39 | (0.36 - 0.42) | 14,072 | 2.29 | (2.08 - 2.51) | 0.32 | (0.28 - 0.36) |
| Middle school graduates | 1,769 | 2.10 | (1.76 - 2.50) | 0.33 | (0.30 - 0.35) | 13,323 | 1.84 | (1.70 - 2.00) | 0.24 | (0.21 - 0.27) |
| High school graduates | 3,282 | 1.59 | (1.35 - 1.87) | 0.25 | (0.23 - 0.27) | 28,241 | 1.39 | (1.31 - 1.48) | 0.19 | (0.16 - 0.21) |
| College graduates | 1,568 | 1.00 |  | 0.16 | (0.13 - 0.18) | 17,751 | 1.00 |  | 0.14 | (0.11 - 0.16) |
|  | 10,582 |  |  |  |  | 73,387 |  |  |  |  |
| Income<200 | 4,126 | 1.42 | (1.29 - 1.57) | 0.33 | (0.31 - 0.36) | 23,752 | 1.57 | (1.45 - 1.69) | 0.25 | (0.21 - 0.28) |
| Income<300 | 1,833 | 1.28 | (1.14 - 1.44) | 0.31 | (0.28 - 0.33) | 15,623 | 1.32 | (1.17 - 1.49) | 0.21 | (0.17 - 0.24) |
| Income <400 | 1,425 | 1.15 | (1.01 - 1.31) | 0.27 | (0.25 - 0.30) | 13,487 | 1.16 | (1.02 - 1.32) | 0.18 | (0.15 - 0.22) |
| Income ≥400 | 3,157 | 1.00 |  | 0.25 | (0.23 - 0.27) | 15,927 | 1.00 |  | 0.16 | (0.14 - 0.18) |
|  | 10,541 |  |  |  |  | 68,789 |  |  |  |  |

K-NHANES: Korea National Health and Nutrition Examination Survey; HEXA-G: Health Examinees-Gem; 95% CI: 95% confidence Interval

^1)^ Enlarged waist circumference ≥ 90 in males and ≥ 85 in females; ^2)^ Sampling weight was adjusted.

Table B. Age-adjusted relative risk (RR) and age-standardized prevalence: Elevated triglycerides ^1)^

|  | K-NHANES^2)^ | | | | | HEXA-G | | | | |
| --- | --- | --- | --- | --- | --- | --- | --- | --- | --- | --- |
|  | N | RR | 95% CI  (lower, upper) | Prevalence | 95% CI  (lower, upper) | N | RR | 95% CI  (lower, upper) | Prevalence | 95% CI  (lower, upper) |
| *Male* |  |  |  |  |  |  |  |  |  |  |
| Elementary school | 1,571 | 0.97 | (0.88 - 1.07) | 0.45 | (0.40 - 0.49) | 3,415 | 0.95 | (0.89 - 1.01) | 0.41 | (0.36 - 0.46) |
| Middle school graduates | 1,351 | 1.01 | (0.92 - 1.11) | 0.45 | (0.41 - 0.49) | 4,896 | 0.98 | (0.93 - 1.04) | 0.39 | (0.36 - 0.42) |
| High school graduates | 2,653 | 0.98 | (0.91 - 1.06) | 0.44 | (0.42 - 0.47) | 13,453 | 0.99 | (0.93 - 1.04) | 0.40 | (0.37 - 0.42) |
| College graduates | 2,276 | 1.00 |  | 0.44 | (0.41 - 0.46) | 16,969 | 1.00 |  | 0.40 | (0.39 - 0.41) |
|  | 7,851 |  |  |  |  | 38,733 |  |  |  |  |
| Income<200 | 2,489 | 0.99 | (0.92 - 1.07) | 0.42 | (0.40 - 0.45) | 9,981 | 0.98 | (0.94 - 1.03) | 0.39 | (0.37 - 0.42) |
| Income<300 | 1,442 | 1.01 | (0.93 - 1.10) | 0.44 | (0.41 - 0.47) | 8,830 | 1.00 | (0.96 - 1.05) | 0.40 | (0.38 - 0.42) |
| Income <400 | 1,212 | 1.07 | (0.99 - 1.17) | 0.47 | (0.44 - 0.50) | 8,005 | 1.02 | (0.98 - 1.06) | 0.41 | (0.40 - 0.42) |
| Income ≥400 | 2,719 | 1.00 |  | 0.43 | (0.41 - 0.45) | 10,124 | 1.00 |  | 0.40 | (0.39 - 0.42) |
|  | 7,862 |  |  |  |  | 36,940 |  |  |  |  |
| *Female* |  |  |  |  |  |  |  |  |  |  |
| Elementary school | 3,813 | 1.79 | (1.52 - 2.12) | 0.33 | (0.30 - 0.36) | 14,084 | 1.31 | (1.25 - 1.37) | 0.26 | (0.24 - 0.28) |
| Middle school graduates | 1,707 | 1.41 | (1.18 - 1.70) | 0.26 | (0.23 - 0.28) | 13,337 | 1.29 | (1.23 - 1.36) | 0.25 | (0.24 - 0.27) |
| High school graduates | 3,198 | 1.36 | (1.16 - 1.61) | 0.24 | (0.22 - 0.26) | 28,271 | 1.17 | (1.13 - 1.20) | 0.22 | (0.21 - 0.23) |
| College graduates | 1,528 | 1.00 |  | 0.18 | (0.15 - 0.21) | 17,766 | 1.00 |  | 0.19 | (0.18 - 0.20) |
|  | 10,246 |  |  |  |  | 73,458 |  |  |  |  |
| Income<200 | 3,944 | 1.38 | (1.24 - 1.54) | 0.29 | (0.27 - 0.31) | 23,781 | 1.21 | (1.16 - 1.26) | 0.25 | (0.23 - 0.26) |
| Income<300 | 1,776 | 1.34 | (1.18 - 1.52) | 0.28 | (0.25 - 0.30) | 15,637 | 1.13 | (1.09 - 1.18) | 0.22 | (0.21 - 0.24) |
| Income <400 | 1,393 | 1.18 | (1.03 - 1.36) | 0.25 | (0.22 - 0.28) | 13,500 | 1.04 | (0.99 - 1.09) | 0.21 | (0.20 - 0.22) |
| Income ≥400 | 3,069 | 1.00 |  | 0.22 | (0.20 - 0.23) | 15,937 | 1.00 |  | 0.20 | (0.19 - 0.21) |
|  | 10,182 |  |  |  |  | 68,855 |  |  |  |  |

K-NHANES: Korea National Health and Nutrition Examination Survey; HEXA-G: Health Examinees-Gem; 95% CI: 95% confidence Interval

^1)^ Elevated triglycerides ≥ 150 mg/dL (1.7 mmol/L) or specific treatment for this lipid abnormality; ^2)^ Sampling weight was adjusted.

Table C. Age-adjusted relative risk (RR) and age-standardized prevalence: Low HDL cholesterol

|  | K-NHANES^2)^ | | | | | HEXA-G | | | | |
| --- | --- | --- | --- | --- | --- | --- | --- | --- | --- | --- |
|  | N | RR | 95% CI  (lower, upper) | Prevalence | 95% CI  (lower, upper) | N | RR | 95% CI  (lower, upper) | Prevalence | 95% CI  (lower, upper) |
| *Male* |  |  |  |  |  |  |  |  |  |  |
| Elementary school | 1,571 | 0.95 | (0.81 - 1.11) | 0.23 | (0.19 - 0.27) | 3,415 | 0.93 | (0.84 - 1.03) | 0.22 | (0.19 - 0.24) |
| Middle school graduates | 1,351 | 0.97 | (0.83 - 1.13) | 0.22 | (0.19 - 0.25) | 4,896 | 0.95 | (0.87 - 1.03) | 0.23 | (0.20 - 0.26) |
| High school graduates | 2,653 | 0.95 | (0.84 - 1.08) | 0.23 | (0.21 - 0.25) | 13,453 | 0.99 | (0.93 - 1.06) | 0.24 | (0.22 - 0.26) |
| College graduates | 2,276 | 1.00 |  | 0.22 | (0.20 - 0.24) | 16,969 | 1.00 |  | 0.24 | (0.22 - 0.26) |
|  | 7,851 |  |  |  |  | 38,733 |  |  |  |  |
| Income<200 | 2,489 | 1.06 | (0.94 - 1.20) | 0.23 | (0.21 - 0.26) | 9,981 | 1.01 | (0.94 - 1.09) | 0.24 | (0.22 - 0.26) |
| Income<300 | 1,442 | 1.08 | (0.94 - 1.24) | 0.24 | (0.21 - 0.26) | 8,830 | 0.99 | (0.94 - 1.04) | 0.23 | (0.22 - 0.25) |
| Income <400 | 1,212 | 1.06 | (0.91 - 1.24) | 0.23 | (0.20 - 0.26) | 8,005 | 1.03 | (0.98 - 1.08) | 0.24 | (0.22 - 0.26) |
| Income ≥400 | 2,719 | 1.00 |  | 0.21 | (0.20 - 0.23) | 10,124 | 1.00 |  | 0.24 | (0.22 - 0.26) |
|  | 7,862 |  |  |  |  | 36,940 |  |  |  |  |
| *Female* |  |  |  |  |  |  |  |  |  |  |
| Elementary school | 3,813 | 1.45 | (1.29 - 1.62) | 0.48 | (0.44 - 0.51) | 14,084 | 1.28 | (1.21 - 1.36) | 0.42 | (0.39 - 0.44) |
| Middle school graduates | 1,707 | 1.26 | (1.12 - 1.42) | 0.41 | (0.38 - 0.44) | 13,337 | 1.23 | (1.16 - 1.30) | 0.41 | (0.39 - 0.42) |
| High school graduates | 3,198 | 1.17 | (1.05 - 1.31) | 0.38 | (0.36 - 0.40) | 28,271 | 1.11 | (1.05 - 1.18) | 0.36 | (0.35 - 0.37) |
| College graduates | 1,528 | 1.00 |  | 0.31 | (0.28 - 0.34) | 17,766 | 1.00 |  | 0.33 | (0.30 - 0.35) |
|  | 10,246 |  |  |  |  | 73,458 |  |  |  |  |
| Income<200 | 3,944 | 1.27 | (1.17 - 1.37) | 0.45 | (0.43 - 0.47) | 23,781 | 1.12 | (1.07 - 1.18) | 0.39 | (0.37 - 0.41) |
| Income<300 | 1,776 | 1.18 | (1.08 - 1.30) | 0.42 | (0.39 - 0.45) | 15,637 | 1.07 | (1.02 - 1.13) | 0.37 | (0.35 - 0.39) |
| Income <400 | 1,393 | 1.09 | (0.98 - 1.21) | 0.38 | (0.35 - 0.41) | 13,500 | 1.02 | (0.97 - 1.08) | 0.35 | (0.33 - 0.37) |
| Income ≥400 | 3,069 | 1.00 |  | 0.36 | (0.34 - 0.38) | 15,937 | 1.00 |  | 0.35 | (0.33 - 0.37) |
|  | 10,182 |  |  |  |  | 68,855 |  |  |  |  |

K-NHANES: Korea National Health and Nutrition Examination Survey; HEXA-G: Health Examinees-Gem; 95% CI: 95% confidence Interval

^1)^ Low HDL cholesterol < 40 mg/dL (1.03 mmol/L) in males, < 50 mg/dL (1.29 mmol/L) in females or specific treatment for this lipid abnormality; ^2)^ Sampling weight was adjusted.

Table D. Age-adjusted relative risk (RR) and age-standardized prevalence: Elevated blood pressure^1)^

|  | K-NHANES^2)^ | | | | | HEXA-G | | | | |
| --- | --- | --- | --- | --- | --- | --- | --- | --- | --- | --- |
|  | N | RR | 95% CI  (lower, upper) | Prevalence | 95% CI  (lower, upper) | N | RR | 95% CI  (lower, upper) | Prevalence | 95% CI  (lower, upper) |
| *Male* |  |  |  |  |  |  |  |  |  |  |
| Elementary school | 1,629 | 1.06 | (0.97 - 1.15) | 0.50 | (0.45 - 0.55) | 3,412 | 1.02 | (0.97 - 1.08) | 0.55 | (0.51 - 0.58) |
| Middle school graduates | 1,382 | 1.03 | (0.95 - 1.12) | 0.48 | (0.45 - 0.52) | 4,894 | 1.04 | (0.98 - 1.11) | 0.53 | (0.48 - 0.57) |
| High school graduates | 2,709 | 1.10 | (1.02 - 1.18) | 0.51 | (0.49 - 0.53) | 13,446 | 1.05 | (1.01 - 1.09) | 0.53 | (0.49 - 0.56) |
| College graduates | 2,307 | 1.00 |  | 0.46 | (0.44 - 0.49) | 16,961 | 1.00 |  | 0.50 | (0.46 - 0.54) |
|  | 8,027 |  |  |  |  | 38,713 |  |  |  |  |
| Income<200 | 2,535 | 1.03 | (0.96 - 1.10) | 0.50 | (0.47 - 0.53) | 9,977 | 1.00 | (0.96 - 1.04) | 0.51 | (0.47 - 0.56) |
| Income<300 | 1,452 | 0.94 | (0.87 - 1.03) | 0.46 | (0.43 - 0.49) | 8,823 | 1.00 | (0.96 - 1.04) | 0.51 | (0.48 - 0.55) |
| Income <400 | 1,210 | 1.03 | (0.94 - 1.12) | 0.50 | (0.46 - 0.53) | 8,000 | 0.98 | (0.95 - 1.02) | 0.51 | (0.47 - 0.55) |
| Income ≥400 | 2,706 | 1.00 |  | 0.49 | (0.47 - 0.51) | 10,121 | 1.00 |  | 0.51 | (0.48 - 0.55) |
|  | 7,903 |  |  |  |  | 36,921 |  |  |  |  |
| *Female* |  |  |  |  |  |  |  |  |  |  |
| Elementary school | 3,954 | 1.90 | (1.65 - 2.18) | 0.44 | (0.40 - 0.47) | 14,074 | 1.47 | (1.37 - 1.57) | 0.45 | (0.42 - 0.48) |
| Middle school graduates | 1,774 | 1.74 | (1.50 - 2.03) | 0.39 | (0.36 - 0.42) | 13,328 | 1.41 | (1.31 - 1.51) | 0.41 | (0.39 - 0.44) |
| High school graduates | 3,274 | 1.46 | (1.28 - 1.67) | 0.33 | (0.31 - 0.35) | 28,258 | 1.27 | (1.21 - 1.33) | 0.36 | (0.34 - 0.38) |
| College graduates | 1,564 | 1.00 |  | 0.24 | (0.21 - 0.27) | 17,758 | 1.00 |  | 0.30 | (0.28 - 0.32) |
|  | 10,566 |  |  |  |  | 73,418 |  |  |  |  |
| Income<200 | 4,072 | 1.18 | (1.09 - 1.27) | 0.39 | (0.36 - 0.41) | 23,765 | 1.28 | (1.21 - 1.35) | 0.40 | (0.37 - 0.43) |
| Income<300 | 1,798 | 1.10 | (1.00 - 1.22) | 0.36 | (0.33 - 0.38) | 15,631 | 1.20 | (1.14 - 1.26) | 0.37 | (0.34 - 0.40) |
| Income <400 | 1,408 | 1.12 | (1.00 - 1.25) | 0.36 | (0.33 - 0.39) | 13,495 | 1.12 | (1.07 - 1.18) | 0.35 | (0.32 - 0.37) |
| Income ≥400 | 3,123 | 1.00 |  | 0.33 | (0.31 - 0.35) | 15,930 | 1.00 |  | 0.32 | (0.30 - 0.34) |
|  | 10,401 |  |  |  |  | 68,821 |  |  |  |  |

K-NHANES: Korea National Health and Nutrition Examination Survey; HEXA-G: Health Examinees-Gem; 95% CI: 95% confidence Interval

^1)^ Elevated blood pressure (BP) systolic BP ≥ 130 or diastolic BP ≥ 85 mm Hg or specific treatment for this hypertension; ^2)^ Sampling weight was adjusted.

Table E. Age-adjusted relative risk (RR) and age-standardized prevalence: Elevated blood glucose^1)^

|  | K-NHANES^2)^ | | | | | HEXA-G | | | | |
| --- | --- | --- | --- | --- | --- | --- | --- | --- | --- | --- |
|  | N | RR | 95% CI  (lower, upper) | Prevalence | 95% CI  (lower, upper) | N | RR | 95% CI  (lower, upper) | Prevalence | 95% CI  (lower, upper) |
| *Male* |  |  |  |  |  |  |  |  |  |  |
| Elementary school | 1,567 | 1.08 | (0.99 - 1.19) | 0.43 | (0.38 - 0.47) | 3,356 | 1.06 | (0.96 - 1.16) | 0.36 | (0.33 - 0.38) |
| Middle school graduates | 1,352 | 1.15 | (1.05 - 1.26) | 0.46 | (0.43 - 0.50) | 4,784 | 1.07 | (0.98 - 1.17) | 0.36 | (0.32 - 0.39) |
| High school graduates | 2,660 | 1.04 | (0.95 - 1.13) | 0.41 | (0.39 - 0.43) | 13,120 | 1.08 | (1.02 - 1.15) | 0.34 | (0.32 - 0.37) |
| College graduates | 2,279 | 1.00 |  | 0.40 | (0.38 - 0.43) | 16,394 | 1.00 |  | 0.32 | (0.28 - 0.36) |
|  | 7,858 |  |  |  |  | 37,654 |  |  |  |  |
| Income<200 | 2,451 | 0.98 | (0.90 - 1.07) | 0.41 | (0.38 - 0.43) | 9,727 | 1.03 | (0.94 - 1.13) | 0.33 | (0.30 - 0.37) |
| Income<300 | 1,416 | 0.99 | (0.90 - 1.08) | 0.41 | (0.38 - 0.44) | 8,597 | 1.02 | (0.92 - 1.12) | 0.33 | (0.30 - 0.36) |
| Income <400 | 1,196 | 1.06 | (0.96 - 1.16) | 0.44 | (0.41 - 0.47) | 7,780 | 1.03 | (0.96 - 1.10) | 0.33 | (0.30 - 0.36) |
| Income ≥400 | 2,673 | 1.00 |  | 0.41 | (0.39 - 0.44) | 9,761 | 1.00 |  | 0.32 | (0.28 - 0.37) |
|  | 7,736 |  |  |  |  | 35,865 |  |  |  |  |
| *Female* |  |  |  |  |  |  |  |  |  |  |
| Elementary school | 3,802 | 1.65 | (1.41 - 1.93) | 0.34 | (0.31 - 0.37) | 13,729 | 1.56 | (1.42 - 1.72) | 0.24 | (0.22 - 0.27) |
| Middle school graduates | 1,710 | 1.45 | (1.23 - 1.70) | 0.29 | (0.27 - 0.32) | 12,974 | 1.38 | (1.26 - 1.51) | 0.20 | (0.18 - 0.22) |
| High school graduates | 3,196 | 1.21 | (1.03 - 1.40) | 0.25 | (0.23 - 0.27) | 27,572 | 1.25 | (1.16 - 1.35) | 0.18 | (0.16 - 0.19) |
| College graduates | 1,527 | 1.00 |  | 0.22 | (0.19 - 0.25) | 17,355 | 1.00 |  | 0.15 | (0.13 - 0.17) |
|  | 10,235 |  |  |  |  | 71,630 |  |  |  |  |
| Income<200 | 3,907 | 1.30 | (1.17 - 1.44) | 0.31 | (0.29 - 0.33) | 23,067 | 1.27 | (1.17 - 1.39) | 0.20 | (0.19 - 0.22) |
| Income<300 | 1,752 | 1.17 | (1.04 - 1.32) | 0.28 | (0.25 - 0.30) | 15,244 | 1.15 | (1.05 - 1.26) | 0.18 | (0.17 - 0.20) |
| Income <400 | 1,379 | 1.10 | (0.96 - 1.26) | 0.27 | (0.24 - 0.30) | 13,192 | 1.07 | (0.98 - 1.16) | 0.17 | (0.16 - 0.19) |
| Income ≥400 | 3,039 | 1.00 |  | 0.25 | (0.23 - 0.27) | 15,545 | 1.00 |  | 0.16 | (0.14 - 0.18) |
|  | 10,077 |  |  |  |  | 67,048 |  |  |  |  |

K-NHANES: Korea National Health and Nutrition Examination Survey; HEXA-G: Health Examinees-Gem; 95% CI: 95% confidence Interval

^1)^ Elevated fasting plasma glucose (FPG) ≥ 100 mg/dL (5.6 mmol/L) or specific treatment for this glucose abnormality; ^2)^ Sampling weight was adjusted.
